# Adaphostin toxicity in a sensitive non-small cell lung cancer model is mediated through Nrf2 signaling and heme oxygenase 1

**DOI:** 10.1186/1756-9966-29-91

**Published:** 2010-07-09

**Authors:** Nicole D Fer, Robert H Shoemaker, Anne Monks

**Affiliations:** 1 Laboratory of Functional Genomics, SAIC-Frederick Inc., NCI-Frederick, (1050 Boyles Street), Frederick, (21702), USA; 2 Sreening Technologies Branch, NCI-Frederick, (1050 Boyles Street), Frederick, (21702), USA

## Abstract

**Background:**

Preclinical toxicity of adaphostin has been related to oxidative stress. This study investigated the regulatory mechanism underlying adaphostin induction of heme oxygenase 1 (HMOX1) which plays a significant role in modulation of drug-induced toxicity in the non-small cell lung cancer cell line model, NCI-H522.

**Methods:**

The transcriptional response of NCI-H522 to adaphostin prominently involved oxidative stress genes, particularly HMOX1. Reactive oxygen species (ROS) involvement was additionally established by generation of ROS prior to modulation of adaphostin-toxicity with antioxidants. To identify up-stream regulatory elements of HMOX1, immunofluorescence was used to evaluate nuclear translocation of the transcription factor, NF-E2-related factor 2 (Nrf2), in the presence of adaphostin. The PI3-kinase inhibitor, wortmannin, was employed as a pharmacological inhibitor of this process.

**Results:**

Generation of ROS provided a substantial foundation for the sensitivity of NCI-H522 to adaphostin. However, in contrast to leukemia cell lines, transcriptional response to oxidative stress was associated with induction of HMOX1, which was dependent on nuclear translocation of the transcription factor, Nrf2. Pretreatment of cells with wortmannin inhibited translocation of Nrf2 and induction of HMOX1. Wortmannin pretreatment was also able to diminish adaphostin induction of HMOX1, and as a consequence, enhance the toxicity of adaphostin to NCI-H522.

**Conclusions:**

Adaphostin-induced oxidative stress in NCI-H522 was mediated through nuclear translocation of Nrf2 leading to upregulation of HMOX1. Inhibition of Nrf2 translocation by wortmannin inhibited this cytoprotective response, and enhanced the toxicity of adaphostin, suggesting that inhibitors of the PI3K pathway, such as wortmannin, might augment the antiproliferative effects of adaphostin in solid tumors that depend on the Nrf2/ARE pathway for protection against oxidative stress.

## Background

Adaphostin (NSC 680410) is the adamantyl ester of tyrphostin AG957 (NSC 654705) and inhibits the p210bcr-abl tyrosine kinase in CML, but is also toxic against cells without the fusion protein[1]. The toxicity of adaphostin against leukemia cells has been shown to require generation of reactive oxygen species (ROS) [2] and involve iron homeostasis [3], and most work on this compound has focused on hematologic malignancies. However, *in vitro *testing of adaphostin in the NCI-60 cell line panel indicated that several solid tumor cancer cell lines also demonstrated considerable sensitivity to adaphostin, indicating there may be a role for adaphostin in treatment of solid tumors. The prostate tumor cell line, PC3 was published as a model to demonstrate signaling cascades involved in adaphostin induced growth inhibition and cell cycle arrest [4], but this cell line is an order of magnitude more resistant than the lung tumor model NCI-H522 to the growth inhibitory effects of the drug in the NCI-60 human tumor cell line screen (data on DTP website: http://dtp.nci.nih.gov/). An early report showed an anti-tumor effect on an orthotopic glioblastoma model U87, in combination with the Flt-1/Fc chimera [5], and more recent evaluation of adaphostin activity in glioblastoma cell lines identified a high level of HMOX1 induction [6]. HMOX1 is the first and rate limiting step in the degradative pathway of heme, but has also been recognized as an integral part of a cytoprotective mechanism against oxidative stress [7,8]. HMOX1 is a target gene of the basic leucine zipper (bZIP) transcription factor, nuclear factor erythroid 2-like 2, Nrf2 (NFE2L2), a central regulator of cellular oxidative stress response and represents an adaptive response that increases cell resistance to oxidative injury. Nrf2 is readily induced in response to ROS through the Nrf2-ARE pathway which transcriptionally up regulates antioxidant genes in order to protect cells [9]. Nrf2 is regulated through PI3K/AKT pathway [10-12], and translocated into the nucleus where it binds to the antioxidant responsive element (ARE) which results in activation of this enhancer element and initiates the transcription of genes encoding phase II detoxification enzymes. These enzymes [8,9] initiate an antioxidant response, which can be beneficial for cancer prevention [13]. However, the Nrf2-ARE pathway has recently been implicated in chemoresistance and the feasibility of Nrf2 inhibition as a strategy for sensitizing cells to chemotherapeutics was demonstrated [13-15]. HMOX1 upregulation has been identified in the adaphostin response in adherent cell lines, but not in hematopoietic cell line models, and it appears that adaphostin activates a different oxidative stress response in solid tumor models than in leukemia models. Thus, we have investigated the mechanism behind HMOX1 induction in the adaphostin-sensitive lung tumor cell line NCI-H522, and demonstrated an enhancement of adaphostin toxicity following inhibition of Nrf2 nuclear translocation with the PI3K inhibitor wortmannin.

## Methods

### Drugs and Cell Culture

Adaphostin (NSC 680410) and wortmannin (NSC 221019) were obtained from the repository of the National Cancer Institute's Developmental Therapeutics Program (Rockville, Maryland). Desferrioxamine (DFX) and N-acetyl-cysteine (NAC) were purchased from Sigma^® ^(St. Louis, Missouri). NCI-H522, and the leukemia cell lines, (Jurkat, HL60 and K562) were obtained from the NCI-60 Human Tumor Cell Line Screen (National Cancer Institute-Frederick, Maryland).

### Transcriptional Profiling: Microarray Technology

Human OperonV2, 20K arrays, (National Cancer Institute microarray facility/Advanced Technology Center, Gaithersburg, Maryland) were utilized according to published protocols http://madb.nci.nih.gov/. Using competitive hybridization of treated versus untreated samples chemically coupled to a Cy™3 or Cy™5 fluorescently labeled dye (Amersham Biosciences, Little Chalfont Buckinghamshire, England) and fluorescence was read on a GenePix 4100A microarray scanner purchased from Axon Instruments (Union City, California). Data was analyzed using the Axon GenePix Pro 4.1 software and data and image files were then uploaded to the National Cancer Institute/Cancer Center for Research Microarray Center mAdB Gateway for analysis and comparison of multiple arrays.

### Real Time RT-PCR

Five hundred nanograms of total RNA for each sample was reverse transcribed using the GeneAmp^® ^PCR System 9700 and TaqMan^® ^Reverse Transcription Reagents kit. Quantitative real time PCR reactions were conducted and measured using the ABI Prism™ 7700 Sequence Detection System and TaqMan^® ^chemistries using published primers. Samples were tested in triplicate wells for the genes of interest and for the endogenous control, 18 S. Data was analyzed using the comparative Ct method as described in the Perkin Elmer User Bulletin #2 (ABI Prism^® ^7700 Sequence Detection System, 1997) and expressed as a fold induction of the gene in the adaphostin treated samples compared to the untreated control samples, and significant differences were calculated using a paired two sample t-test.

### Western Blot

Whole cell and nuclear extracts were made for protein analysis by western blot. Nuclear extracts were prepared from cells in 100 mm dishes that were lysed using a hypotonic buffer. The nuclei were pelleted at 13,000 × g for 15 minutes, and then after the supernatant was aspirated, the nuclei were lysed using 1x RIPA lysis buffer (Upstate, Lake Placid, New York) containing protease inhibitors (Roche, Mannheim, Germany). Protein was quantitated using Bradford Protein Assay (Bio-Rad Laboratories, Hercules, California), and approximately 50 μg of each sample was resolved by SDS-PAGE on 10% Tris glycine gels (Invitrogen, Carlsbad, California) and probed with anti-Nrf2 (Santa Cruz Biotechnology, Santa Cruz, California) and anti-HMOX1 antibodies (Affinity BioReagents, Golden, Colorado). Proteins were visualized using chemiluminescence and imaged using a Kodak™ X-OMAT 2000A Processor (Rochester, New York).

### Measurement of adaphostin-induced ROS

Intracellular ROS were measured after 2 and 4 hours exposure to 1 μM adaphostin using 2',7'-dichlorofluorescein diacetate (DCFH-DA, Sigma^®^, St. Louis, Missouri). Cells were incubated for 3 minutes with 10 μM DCFH-DA, lysed and centrifuged. The fluorescence was read on a Wallac Victor 2 I420 Multilabel Counter (PerkinElmer, Waltham, Massachusetts) at excitation of 485 nm and emission of 535 nm and protein normalized using Bradford Protein Assay. Results were expressed as percentage increase compared to control and significant differences calculated using a two sample t-test assuming equal variances.

### Modulation of growth inhibition

Cells were inoculated onto 96 well plates (20,000 cells/well) and preincubated with DFX (100 μM), NAC (25 mM) or wortmannin (250 nM) prior to addition of adaphostin for a further 96 h incubation. Growth inhibition was assessed by alamarBlue (Sigma^®^, St. Louis, Missouri), fluorescence was read on a Tecan Ultra plate reader (509 nm excitation and 520 nm emission); and results analyzed using the average percent treated/control (%T/C), with significant differences calculated using a paired two sample t-test.

### Immunofluorescence

Cells were plated in Lab-Tek chamber slides (60,000 cells/well) and treated 4-6 hours with 1 μM adaphostin, or pretreated 30 minutes with 500 nM wortmannin, followed by 4 hour incubation with 1 μM adaphostin where indicated. Cells were fixed using cold methanol; permeabilized with 0.1% Triton X-100; blocked in 20% goat serum; incubated with Nrf2 antibody overnight; labeled using FITC-conjugated secondary antibody; and nuclei were counter-stained with DAPI. Prolong Anti-Fade (Invitrogen, Carlsbad, California) was used to mount coverslip overnight. Samples were visualized using a Leitz Laborlux D fluorescence microscope and images were captured by Leica DFC420 camera and analyzed in Adobe Photoshop Elements 2.0.

## Results

**Figure 1:**
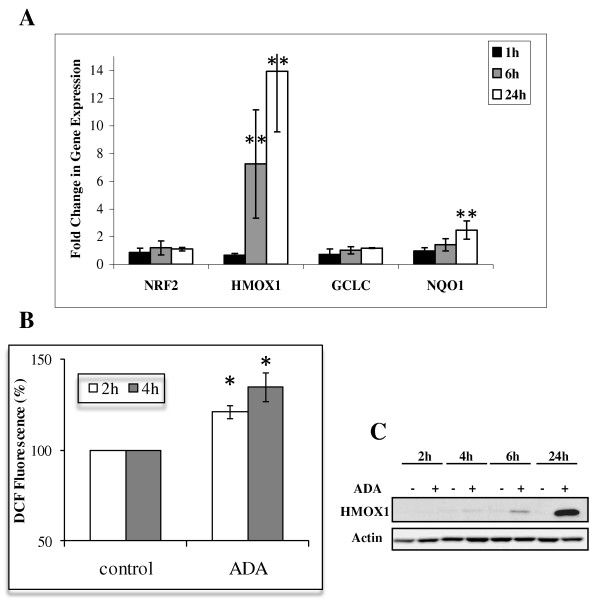
**Adaphostin (ADA) effect on HMOX1 related genes, ROS, and HMOX1 protein**. **(A) **ADA modulation of NRF2, HMOX1, GCLC, and NQO1 gene expression. Cells were treated with 1 μM of ADA for 1, 6 and 24 h and gene expression was measured by microarray and quantitative RT/PCR and expressed as fold change of drug -treated NRF2, HMOX1, GCLC, and NQO1 compared with control (n = 4; +/- SD). Both HMOX1 and NQO1 were significantly up-regulated by ADA (** p < 0.01). **(B) **Increased ROS production after ADA treatment. Cells were treated for 2 and 4 h with 1 μM ADA and ROS was measured using DCFH-DA (10 μM). There was a significant increase in ADA-induced ROS production. After 2 and 4 h (n = 2 +/- SD, * p < 0.05). **(C) **ADA induces HMOX1 protein. NCI-H522 cells were incubated for 2 h, 4 h and 6 h with 1 μM of ADA and whole cell extracts were resolved by Western blot analysis as indicated in the Materials and Methods. Data are representative of three independent experiments.

**Figure 2:**
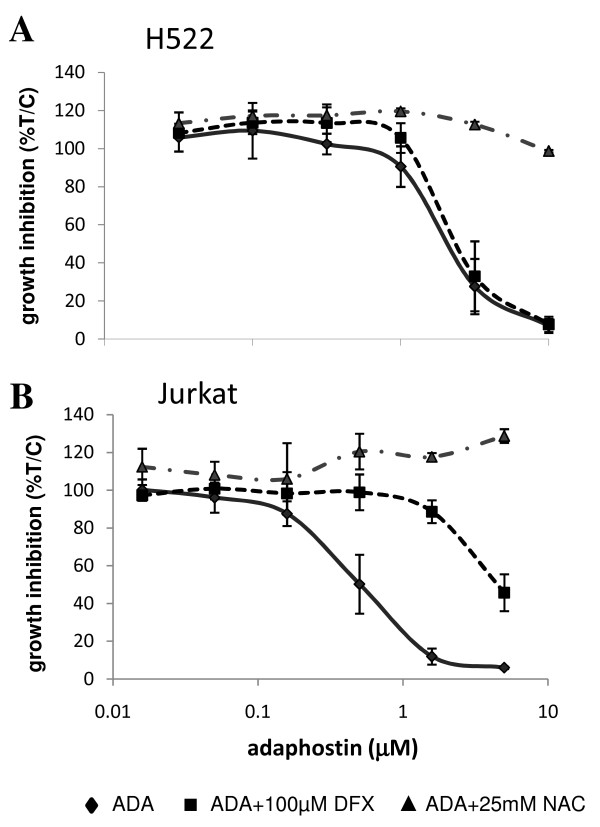
**The presence of ROS is an important factor in determining sensitivity to adaphostin (ADA)**. **(A) **Dose response curves of NCI-H522 after treatment with ADA either alone or in combination with 25 mM n-acetyl cysteine (NAC) or 100 μM desferrioxamine (DFX). ADA sensitivity was attenuated by NAC, but not DFX (n = 3; +/- SD). **(B) **Dose response curves of Jurkat after treatment with ADA either alone or in combination with 25 mM NAC or 100 μM DFX. ADA sensitivity was attenuated by NAC and DFX (n = 3; +/- SD).

Although hematopoietic malignancies have been the major target of pre-clinical studies with adaphostin [1-3,5,16-19], NCI-H522, a solid tumor-derived, non-small lung cancer cell line, was also very sensitive to adaphostin in the NCI-60 human tumor cell line screen. From four independent experiments in the NCI-60 screen, the 50% growth inhibitory concentration (GI50) for the 6 leukemia cell lines ranged from 40 nM -630 nM, and the GI50 for NCI-H522 was 79 nM, which was 10-fold more sensitive than the average response for the whole cell line panel (762 nM) (data available at: http://dtp.nci.nih.gov/ for NSC 680410). Transcriptional profiling of NCI-H522 in response to 1 μM adaphostin showed one of the most highly upregulated genes to be HMOX1 (11.3 +/- 2.1 (SD) fold increase after 24 h), which encodes for an enzyme that protects against oxidative stress [7,8]. This increase in HMOX1 expression was confirmed using Q-RT/PCR which also corroborated the lack of significant change in expression of the NRF2 gene (figure 1A). Moreover, a small but significant increase in the Nrf2 transcriptional target gene, NAD(P)H dehydrogenase, quinone 1 NQO1 was observed although there was no change in another Nrf2 target, the catalytic subunit of glutamate-cysteine ligase GCLC (figure 1A). A significant increase in ROS production was observed as early as 2 h after adaphostin treatment which is confirmation of the presence of drug-induced oxidative stress (figure 1B). Heme oxygenase 1, the protein encoded by HMOX1, was shown to be increased by adaphostin treatment (1 μM) at a later time point than HMOX1, being only slightly increased after 6 h, but highly expressed after 24 h (figure 1C). These data are consistent with the 10 μM adaphostin-induced heme oxygenase 1 expression reported in glioblastoma cell lines, which did not appear until after 8-24 h [6]. This adaphostin-induced HMOX1 upregulation in NCI-H522 cells and glioblastoma cell lines [6] is in contrast to the response of hematologic cell lines where we have previously reported the major transcriptional response involved >10-fold induction of genes encoding for both heavy and light ferritin polypeptides (FTH and FTL) [3]. Moreover, even after treatment with 10 μM adaphostin, leukemia cell lines (Jurkat, HL60 and K562) showed no increase in HMOX1 expression on the cDNA arrays after 6 h incubation (average expression (n = 3) = 1.24 +/- 0.7(SD), 1.35 +/- 0.39(SD) and 1.16 +/- 0.28(SD) respectively), compared to a 7.4 and 30.8 -fold increase in HMOX1 expression in NCI-H522 cells when measured on the same type of arrays following treatment with 1 and 4 μM adaphostin for 6 h. Evidence that ROS are an important factor in determining sensitivity of NCI-H522 to adaphostin was demonstrated by the ablation of adaphostin toxicity by the anti-oxidant, N-acetyl-cysteine in a manner similar to that shown for the leukemia cell line Jurkat (figure 2). However, in contrast to Jurkat, the iron chelating agent desferrioxamine (100 μM) did not attenuate adaphostin toxicity in the NCI-H522 cell line (figure 2), and there was no measurable increase in either ferritin gene (array expression (n = 5): FTH = 1.09 +/- 0.15 (SD); FTL = 1.02 +/- 0.24(SD)), indicating that release of excess free iron is not involved in the NCI-H522 response to adaphostin. Thus, these data substantiate the difference between response of a solid tumor and that which we have shown in leukemia cell lines [3].

**Figure 3:**
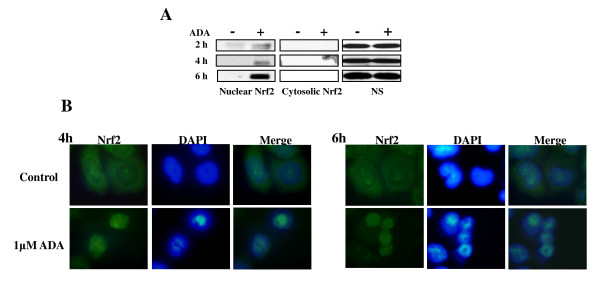
**Adaphostin (ADA) induces nuclear localization of Nrf2 protein**. **(A) **NCI-H522 cells were incubated for 2 h, 4 h and 6 h with 1 μM of ADA; nuclear and cytosolic proteins were resolved by Western blot analysis as indicated in the Materials and Methods (NS, non-specific band was used for normalization). Data are representative of three independent experiments. **(B) **Confirmation of ADA-induced Nrf2 translocation into the nucleus. NCI-H522 cells were incubated for 4 h or 6 h with 1 μM ADA and were stained with Nrf2 and FITC-conjugated antibodies. Nuclei were counter-stained with the fluorescence dye DAPI. Data are representative of three independent experiments.

As the induction of HMOX1 appears to be unique to the response of solid tumors [6], we investigated the role of its putative regulatory transcription factor, Nrf2, in adaphostin treated NCI-H522 cells. Nrf2 protein, when activated is rapidly translocated into the nucleus, and in adaphostin-treated NCI-H522 cells, Nrf2 was rapidly induced in the nuclear fraction within 2-6 h, although there was no detectable Nrf2 expression in the cytosolic fraction over this time (figure 3A). Furthermore, translocation of Nrf2 from the cytoplasm into the nucleus by adaphostin can be visualized using immunohistochemistry (figure 3B) where nuclear localization of Nrf2 after 4 h and 6 h incubation of NCI-H522 cells with 1 μM adaphostin was apparent compared to the more diffuse Nrf2 distribution in untreated cells.

**Figure 4:**
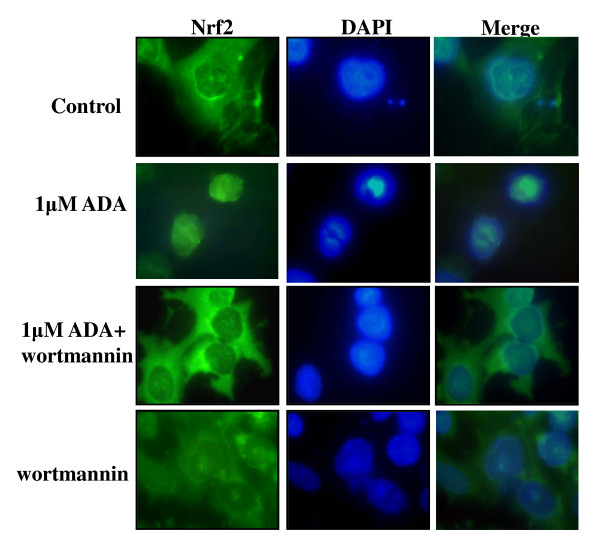
**Wortmannin inhibits adaphostin (ADA)-induced translocation of Nrf2 into the nucleus**. NCI-H522 cells were pretreated 30 minutes with 500 nM wortmannin where indicated, followed by 4 hour incubation with 1 μM ADA and stained with Nrf2 and FITC-conjugated antibodies. Nuclei were counter-stained with the fluorescence dye DAPI. Data are representative of two independent experiments.

**Figure 5:**
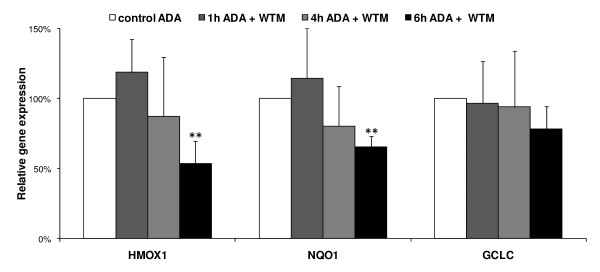
**Adaphostin (ADA) induction of HMOX1 and NQO1 is inhibited by the presence of wortmannin (WTM)**. NCI-H522 cells were pretreated 30 minutes with 500 nM WTM, followed by incubation with 1 μM ADA. Expression of HMOX1, NQO1 and GCLC was measured by quantitative real-time reverse transcription-PCR after a further 1, 4 and 6 h and expressed as a percentage of the control ADA-induced gene expression measured at that time in the absence of WTM pretreatment. There was a significant decrease in 6 h ADA-induced HMOX1 and NQO1expression after wortmannin pretreatment (n = 3; +/- SD; ** indicates p < 0.01)

Wortmannin, a PI3 kinase inhibitor, has been shown to inhibit Nrf2 translocation into the nucleus [20,21] and was successfully used as a tool to inhibit adaphostin-induced, nuclear translocation of Nrf2 (figure 4). Pretreatment (30 minutes) of NCI-H522 cells with 500 nM wortmannin was effective at inhibiting adaphostin-induced nuclear localization of Nrf2, although wortmannin alone had no effect. In addition, under these conditions when Nrf2 translocation was inhibited with wortmannin, expression of Nrf2 target genes HMOX1 and NQO1were significantly (p < 0.01) reduced by ~50% and ~35% respectively after 6 h adaphostin incubation, and though not significant, there was a trend to a reduced expression after 4 h incubation (figure 5). There was no significant change in GCLC expression which is consistent with the lack of induction of this gene with adaphostin, and implicates Nrf2 as the regulator of adaphostin-induced HMOX1.

**Figure 6:**
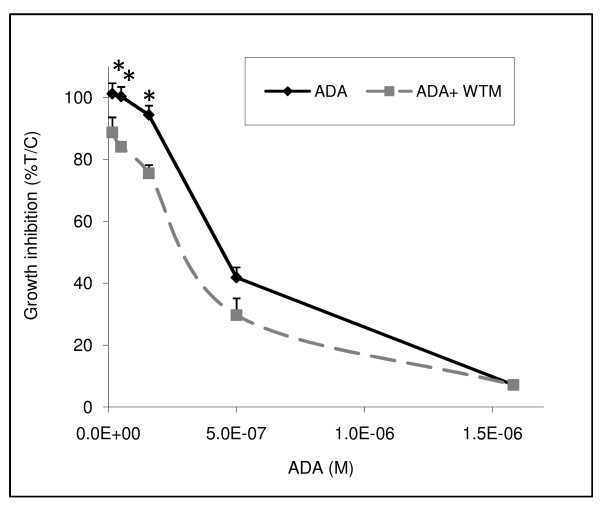
**Adaphostin (ADA) toxicity is enhanced when HMOX1 induction is diminished via inhibition of Nrf2 nuclear translocation by wortmannin**. NCI-H522 cells were pretreated with 250 nM wortmannin, followed by treatment with ADA for an additional 96 h and growth inhibition was assessed with alamarBlue vital dye (n = 4; +/- SD; * indicates p = or <0.01).

Finally, figure 6 shows that when HMOX1 induction was diminished via inhibition of Nrf2 nuclear translocation, there was an augmentation of adaphostin toxicity with a reduction of the GI_50 _from 342 nM to 273 nM, with the most significant effect (p < 0.01) at the lower concentrations of adaphostin.

## Discussion

Adaphostin, is a tyrphostin-like kinase inhibitor whose toxicity to tumor cell lines is a function of its ability to induce oxidative stress and cause a redox imbalance in cells [2,22-25]. In hematologic tumor cell lines, we have previously shown that iron homeostasis and up-regulation of ferritin genes were an integral part of the response to adaphostin [3]. In contrast, evaluation of the transcriptional response of a solid tumor derived, non-small cell lung cancer cell line, NCI-H522, which is equally sensitive to adaphostin as the hematologic cell lines indicated that the HMOX1 gene was the most highly up-regulated gene, and there was very little modulation of the ferritins. The up-regulation of HMOX1 in solid tumor derived models, is consistent with data published for glioblastoma cell lines [6] suggesting that these cell lines may utilize different pathways to handle the adaphostin induced oxidative stress. Moreover, the growth inhibitory curve of adaphostin in NCI-H522 was completely ablated by pretreatment with the antioxidant NAC, but not with desferrioxamine indicating that despite the role of HMOX1 in generating free iron from heme, iron homeostasis is not an important feature of the response to ROS generated by adaphostin. HMOX1 is a stress-inducible enzyme that is most commonly regulated by the basic leucine zipper transcription factor Nrf2, which is a regulator of multiple antioxidant genes [12]. Dramatic induction of HMOX1 appears to be stimulated by adaphostin in this cell line. Another well documented target of Nrf2, NAD(P)H dehydrogenase, quinone 1 (NQO1) was also induced to a lesser extent but there was no evidence for regulation of gamma-glutamylcysteine synthetase (GCLC), which is consistent with data from cultured RPE cells where modulation of Nrf2 activity led to selective down regulation of only certain phase 2 detoxification genes, and not all stimuli resulted in all genes being modulated [11].

Adaphostin triggered the translocation of Nrf2 protein into the nucleus, as measured both by an increase in nuclear protein and immunofluorescence. Nrf2 translocation into the nucleus has been shown to be prevented by the PI3 kinase inhibitor, wortmannin [11,21]. Pretreatment with wortmannin was clearly able to reduce adaphostin-induced Nrf2 nuclear translocation in NCI-H522, and there was a significant decrease in HMOX1 induction after 6 h adaphostin treatment. Thus, these data confirm in a sensitive solid tumor model, NCI-H522, that the major cause of adaphostin toxicity was through generation of ROS, which is the widely accepted model of toxicity for hematologic malignancies [2,3,25]. However, unlike hematologic malignancies, adaphostin initiated an antioxidant response in NCI-H522 cells through up-regulation of HMOX1. The transcriptional increase was initiated through Nrf2, following its translocation into the nucleus, and could be inhibited by wortmannin, implicating the PI3K pathway in the activity of adaphostin. Nrf2 has been identified as a master redox switch involved in the activity of cytoprotective phytochemicals with chemopreventive activity against cancer [26], and plays an important role in the defense against oxidative stress [27]. However, a 'dark side' of Nrf2 has recently been recognized [15], identifying it as responsible for resistance against chemotherapy, thus making Nrf2 a potential target to improve activity of certain chemotherapeutic agents [13,28,29].

## Conclusions

Targeting of the Nrf2 transcription factor may be important for drugs whose major mechanism of action was through the generation of ROS (e.g. adaphostin), as there is evidence for a selective killing of tumor versus normal cells [30], and inhibition of the antioxidant, protective role of Nrf2 may increase the toxic potential of such agents. When NCI-H522 cells were preincubated with wortmannin to inhibit Nrf2 translocation, there was a significant increase in adaphostin toxicity. This data may provide a rationale for successful combinations of adaphostin, or other pro-oxidant agents, with inhibitors of the PI3K pathway as modulators of Nrf2 antioxidant activity.

## Competing interests

The authors declare that they have no competing interests.

## Authors' contributions

NDF was responsible for all experimental data and helped draft the manuscript. RHS aided coordination of the study and helped draft the manuscript. AM conceived of the study, participated in its design and drafted the manuscript. All authors read and approved the final manuscript.
